# Vocal Pitch Discrimination in Children with and without Vocal Fold Nodules

**DOI:** 10.3390/app9153042

**Published:** 2019-07-28

**Authors:** Elizabeth S. Heller Murray, Anne F. Hseu, Roger C. Nuss, Geralyn Harvey Woodnorth, Cara E. Stepp

**Affiliations:** 1 Department of Speech, Language and Hearing Sciences, Boston University, Boston, MA 02215, USA; 2 Department of Otolaryngology and Communication Enhancement, Boston Children’s Hospital, Boston, MA 02115, USA; 3 Department of Otolaryngology—Head and Neck Surgery, Boston University School of Medicine, Boston, MA 02118, USA; 4 Department of Biomedical Engineering, Boston University, Boston, MA 02215, USA

**Keywords:** pitch, voice, vocal fold nodules, auditory discrimination

## Abstract

Vocal pitch discrimination abilities were compared in sixteen children with vocal fold nodules (CwVN) and sixteen matched controls with typical voices (CwTV). Vocal pitch discrimination was also evaluated in thirty-five vocally healthy children and twenty adults to examine potential changes as a function of maturation. CwTV were categorized as either younger (N = 15, 5.6–7.7 years) or older (N = 20, 8.2–11.7 years). Participants completed two-alternative, forced choice listening tasks in which they judged whether pairs of sustained /α/ tokens were different in pitch. Each pair consisted of a base token with a fundamental frequency *f_o_*) of 216.2 Hz and a test token with a *f_o_* that was adaptively modified, according to the participant’s prior judgments. There were no significant differences in pitch discrimination abilities between CwVN and CwTV. Pitch discrimination abilities were significantly poorer in younger and older CwTV as compared to adults. Additionally, younger CwTV had significantly poorer discrimination abilities than older CwTV. Findings from this study suggest that CwVN do not have differences in pitch discrimination abilities, yet, therapies designed for CwVN should consider this developmental trend in perceptual abilities.

## 1. Introduction

The presence of a dysphonic, or altered, vocal quality can have a significant negative impact on a child [1-3]. The most common cause of dysphonia in children is vocal fold nodules, benign, callous-like lesions on the vocal folds [4-11]. Vocal fold nodules are suggested to be related to vocal use, vocal misuse, and/or vocal hyperfunction [6,12-17]. Voice therapy is considered the best practice for the treatment of vocal fold nodules in children [18-22], yet it yields varied success rates [23,24]. This is likely due to multiple factors, including: Lack of transparency with regard to therapy, differing outcome measures, lack of understanding of the exact etiology of vocal fold nodules’ development, and an overall shortage of pediatric-focused research within the greater field of voice disorders [25-27].

A recent study has provided a more comprehensive view of pediatric-focused voice therapy by conducting a randomized clinical trial with children with vocal fold nodules (CwVN) ages 6–10 [28]. Authors selected two therapy approaches to compare: (1) ‘Indirect’ therapy, focusing on vocal hygiene and discussion of desired vocal productions, and (2) ‘direct’ therapy, focusing on motor learning tasks. Results indicated that both therapy approaches resulted in improvement of the primary outcome measure, the Pediatric Voice-Related Quality of Life (PVRQOL) [29]. Improvements of secondary outcome measures, including auditory-perceptual evaluation of voice, vocal fold nodule size, acoustic measures, and aerodynamic measures were also shown. Although no significant differences were found between the two therapy types, *post hoc* analyses indicated that older children (8–10 years) had a larger improvement in their PVRQOL scores for the ‘direct’ therapy, as compared to ‘indirect’ therapy. Authors suggested that these older children may be more cognitively able to learn the new motor patterns during the ‘direct’ therapy sessions and, thus, displayed greater improvements [28].

An alternate (or perhaps, extended) hypothesis for differences seen between older and younger children [28] may be that older children have more developed auditory-perceptual abilities, thus allowing them to better detect, learn, and implement vocal changes. Explicit examination of auditory-perceptual discrimination has not been examined in CwVN, yet previous works suggest that CwVN may have perceptual differences as compared to children without voice disorders [30,31]. Historically, methods of voice therapy for children suggested spending extensive time in therapy on attention and awareness (e.g., [32]), as the supposition was these children were not noticing their own vocal characteristics. More recently, a study examining the parental perception of personality traits suggested that CwVN may have difficulties using detection of small changes as a self-regulation tool, as compared to children without diagnosed speech disorders and children who stutter [31]. Finally, emerging evidence in adults with dysphonic voices indicates that they have poorer pitch discrimination than vocally healthy controls [33]. Overall, these previous studies suggest that explicit examination of auditory-perceptual abilities in CwVN is needed to determine if there are perceptual differences in this population.

Studies examining auditory-perceptual discrimination, typically using pure tones, show that most children do not reach adult-like maturity until at least eight years of age [34-40]. Yet it remains unknown whether mature auditory discrimination is required to efficiently learn and apply new therapy tasks and strategies. Additionally, the ecological validity of evaluating auditory discrimination abilities of pure tones is low, making it difficult to translate these findings to auditory discrimination of changes in the voice. Specifically, pure tones are comprised of a single frequency, whereas the voice has a complex harmonic structure, containing a fundamental frequency as well as multiples of that frequency (i.e., harmonics). To date, only a few studies have examined children’s abilities to discriminate pitch stimuli with more complex stimuli, with findings indicating variable results as to when children become adult-like [40,41]. As such, continued and well-controlled work is necessary to further understand whether discrimination of complex stimuli, such as the voice, changes throughout maturation.

Previous work has discussed the optimal methods of testing auditory processing across the lifespan, with researchers noting that an ideal task is one that requires minimal memory and attentional demands, as those can vary greatly in certain age and/or clinical populations [42]. Additionally, the language used to elicit a response can impact the results: Complex relational language, such as ‘higher’ or ‘lower’ has been shown to result in poorer discrimination as compared to less complex language, such as ‘same’ and ‘different’ [43]. Additionally, attentional abilities of the participant may impact discrimination abilities [35], especially in procedures that have increased attentional demands [36,41,44]. The potential effects of attention on auditory discrimination abilities are especially important to consider when evaluating CwVN, given their personality factors and characteristics discussed in the literature. Specifically, CwVN may also have differences in awareness and attention [45,46]. Lee and colleagues [47] conducted a systematic review to examine personality traits, psychological factors, and behavioral factors that may be related to CwVN. Differing results were found regarding inattentive behaviors;some studies noted that children with voice disorders had higher scores of distractibility [45] and higher parental and teaching ratings of inattentiveness [46]; other studies showed no differences from matched controls [48,49]. Thus, when examining auditory discrimination abilities in children, the experimental methods must be carefully chosen so that potential discrimination differences are not overshadowed by attentional differences.

The current study examined the ability to discriminate vocal pitch using an experimental paradigm with low memory, attention, and language demands. Vocal pitch was selected because it is a salient feature of voice that does not require extensive or complex training to understand. The first hypothesis was that school-age CwVN would have poorer discrimination abilities compared to age- and sex-matched children with typical voices (CwTV), suggestive of a perceptual deficit in CwVN. The second hypothesis was that children under eight years of age would have poorer auditory discrimination abilities compared to older children and adults, similar to trends noted in previous work performed with pure tone stimuli [34-39].

## 2. Materials and Methods

### 2.1. Participants

Fifty-one children (5.6–12.4 years) and 20 adults (19–28 years) participated in the current study. Children over 7.0 years of age provided verbal assent, and dissent was respected for children under 7.0 years of age. For all children in the study, guardians provided written consent. All adult participants provided written consent. Consent and assent were obtained in compliance with the Boston University Institutional Review Board (protocol number: 2625) and were approved by the Boston Children’s Hospital Institutional Review Board. All participants were compensated for participation and spoke English as their primary language. Additionally, as the current study was examining pitch discrimination abilities, none of the included participants spoke or heard tonal languages at home.

### 2.2. Children with Vocal Fold Nodules (CwVN)

Sixteen male CwVN (mean (M) = 9.6 years; range 7.2–12.5 years) were diagnosed with vocal fold nodules using endoscopic examination by a board-certified laryngologist at Boston Children’s Hospital. The typical population that arrives at the clinic for a voice evaluation is male [50]; therefore, this cohort was selected for the current study. CwVN had an average ‘overall severity’ score on the Consensus Auditory-Perceptual Evaluation of Voice (CAPE-V) [51] of 47.6 (range: 34.2–60.4) as rated by a speech-language pathologist blinded to the participants’ group. Two participants were receiving ongoing speech or language services, one was receiving voice therapy, and one had received speech or language services in the past. At the time of their clinical visits, all participants also received a complete visual examination of their ears by a medical professional as part of routine care to evaluate for potential otological issues. No participants reported having hearing concerns at home or at school, no participants were found to have middle ear disease, and no participants were referred for an audiology follow-up after their appointment. Data collection for 14 of the participants was completed at Boston Children’s Hospital clinic in a quiet examination room. The two remaining participants completed the experiment in a sound-attenuated booth at Boston University and passed an audiometric hearing screening at a threshold of 25 dB hearing level (HL) or better, at the frequencies 0.25 kHz, 0.5 kHz, 1 kHz, 2 kHz, 4 kHz, 6 kHz, and 8 kHz.

### 2.3. Children with Typical Voices (CwTV)

Sixteen male age-matched CwTV (M = 8.6; range = 6.8–11.7 years; average difference from CwVN = 12.5 months) without voice complaints were recruited to test our first hypothesis that CwVN would have poor discrimination abilities than CwTV. Voice recordings were available for seventeen of the matched CwTV; participants had an average ‘overall severity’ score on the CAPE-V of 16.1 (range: 0–50.7) rated by a speech-language pathologist blinded to the participant’s group. To address the second hypothesis that younger CwTV would have poorer auditory discrimination abilities than older CwTV, nineteen additional CwTV were recruited (for a total 35 CwTV). The 35 CwTV were subsequently divided into a younger (N = 15; M = 6.9 years; range 5.6–7.7 years; 10 male, 5 female) and an older (N = 20; *M* = 9.5 years; range 8.2–11.7 years; 9 male, 6 female) group. No participants in the CwTV group had received speech or language services within the past year. Four participants had a previous history of speech or language services. Twenty-two participants were recorded at Boston University in a sound-treated booth and passed an audiometric hearing screening at a threshold of 25 dB HL or better, at the frequencies 0.25 kHz, 0.5 kHz, 1 kHz, 2 kHz, 4 kHz, 6 kHz, and 8 kHz. The remaining thirteen participants were recorded offsite in a quiet room at the participants’ homes, and they were unable to participate in a hearing screening. Two children had relevant surgical histories; one received ear tubes and one had an ear wax removal procedure. Parents of all participants reported no current hearing concerns.

### 2.4. Adults

Twenty vocally healthy adults (M = 21.2 years, range 19–28 years, 10 male, 10 female) comprised an adult control group. One participant reported receiving speech or language services as a child; no other participants reported a history of speech, language, or hearing disorders. Participants were recorded at Boston University in a sound-treated booth. All participants passed an audiometric hearing screening at a threshold of 25 dB HL or better at the frequencies 0.25 kHz, 0.5 kHz, and 1 kHz in both ears. For the frequencies 2 kHz, 4 kHz, 6 kHz, and 8 kHz, 13 participants passed a hearing screening at 25 dB HL or better, while the remaining two participants passed at 30 dB HL or better.

### 2.5. Stimuli Development and Presentation

The base token used in the study was constructed from an /α/ production by a single male child (9.9 years old) who was not involved in the current study. Acoustic analysis of the selected token was completed in Praat [52] and indicated that average fundamental frequency (*f_o_*: 216.2 Hz), jitter (local, 0.37%), shimmer (local, 3.92%) and cepstral peak prominence (17.45 dB) were within the normative range for age and gender [53,54]. A certified speech-language pathologist, blinded to the source of the sample, provided an ‘overall severity’ score of 3 on the CAPE-V, indicating normal vocal quality. The sample was cropped to be 500 milliseconds (ms) in duration from the middle of the /α/. A cosine window with a rise and fall time of 50 ms was added to the beginning and end of the stimulus to avoid the perception of unnatural clicking. The token was played to participants through Sennheiser HD 280 Pro headphones (Sennheiser electronic GmbH and Co., KG, Wedemark, Germany). Participants recorded at Boston University completed their study in a sound-treated booth, with experimental manipulation of pitch performed in near real-time by the Eventide Eclipse hardware (Eventide Inc., Little Ferry, NJ, USA). The Eventide Eclipse performed a full-spectrum shift of the sustained /α/, in which the values and spacing of the harmonics were shifted up [55]. This shift changes the *f_o_* of the production, thereby changing the perception of pitch. Participants recorded offsite were presented with previously created pitch shifted stimuli, created with the Eventide Eclipse hardware, on a laptop computer (Lenovo ThinkPad X1 Carbon). Regardless of the equipment used, stimuli were presented at approximately 60–70 dB SPL using a custom MATLAB script (version 8.2; The MathWorks, Inc., Natick, MA, USA).

### 2.6. Experimental Design

#### 2.6.1. Training

Prior to the experiment, all participants underwent training to ensure they understood the paradigm. Participants were presented with two pairs of sequential pure tones; one pair had two identical 440 Hz tones, and the other pair had one tone at 440 Hz and the second tone at 800 Hz (a difference of 10 semitones (ST) between tones). After each pair, participants were asked if the tones were the same or different. If participants answered incorrectly, they did not proceed on to the experimental portion and were excluded from the study. If they successfully completed the training, participants continued on to the experiment.

#### 2.6.2. Experimental Paradigm

**Figure 1.**
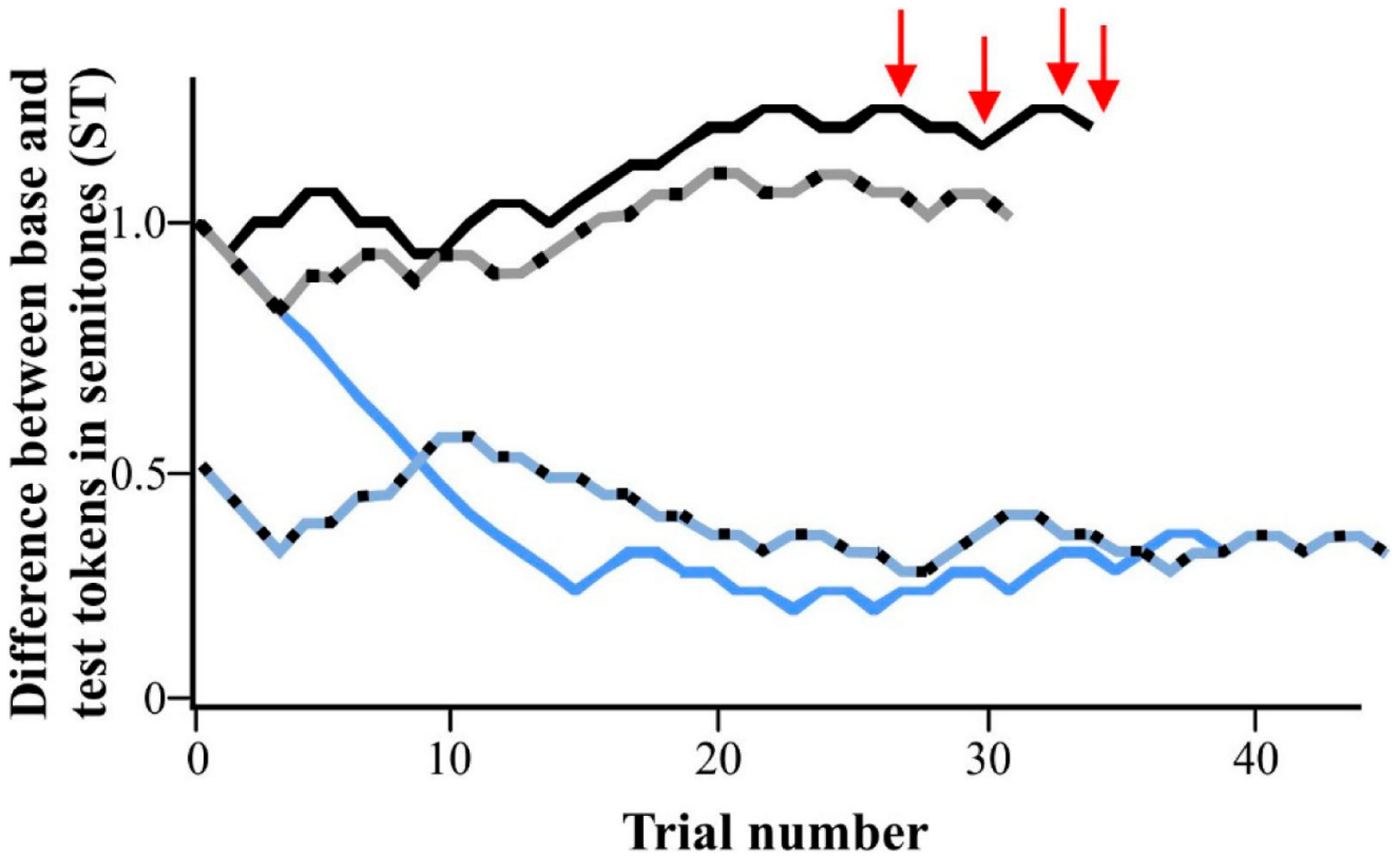
Experimental runs from two participants. The first participant started both run #1 (solid black line) and run #2 at 1 semitone (ST, dotted black line). The second participant started run #1 at 1 ST (solid blue line) and started run #2 at 0.5 ST (dotted blue line). Red arrows indicate the last four reversals that were calculated for the just-noticeable-difference (JND) for participant #1, run #1.

Participants completed a two-alternative forced choice (TAFC) [56] perceptual task in which they judged whether the *f_o_* of two tokens was the ‘same’ or ‘different’ for each trial. The average length of a usable task, hereafter called a run, for children was 5.16 min and the average length for adults was 4.17 min. Tokens presented during each trial were developed from a single /α/ production described in the Stimuli Development and Presentation section. During a single trial, participants heard a 500 ms, unmodified base token (average *f_o_* of 216.2 Hz) and a 500 ms test token that was either (1) the same unmodified base token (216.2 Hz) or (2) a token with a modified*f_o_*. The timing between the two tokens in a single trial was 500 ms. Following the presentation of both tokens, the participant stated whether the two tokens were the same or different and the experimenter advanced to the next trial. Token order (i.e., whether the base token was presented first or second) was randomly determined for each trial. Approximately one-third of the trials had two tokens with the same*f_o_*, called ‘catch’ trials. These ‘catch’ trials were used to ensure data validity, attention to the task, and understanding of task instructions. The remaining trials were ‘different’ trials, in which the *f_o_* value of the test token was adaptively altered based on the participant’s performance, using the stair-step procedure outlined by Levitt [57]. All adults began with a test token that was 0.5 ST, that is, 6.34 Hz, higher than the base token. Based on the known developmental trends reported in the literature for pitch discrimination with pure tones (e.g., 34, 37–40]), the test token was set between 0.5–3 ST (6.34–40.91 Hz) higher than the base token for the children. For each child, the experimenter determined the starting value of the test token according to the child’s age as well as compliance during the training. Prior to implementing the flexible starting position, pilot testing indicated that using a variable starting place did not preclude an individual from reaching an estimate of their pitch discrimination abilities (Figure 1).

Step-size magnitude was defined as the magnitude of change in *f_o_* during the adaptive procedure. The first 10 trials had a step-size of 0.1 ST and the remaining trials had a smaller step-size of 0.06 ST, regardless of the direction of the change. The experimental paradigm began with a 1-down-1-up TAFC paradigm, wherein the *f_o_* of the test token was moved closer to the *f_o_* of the base token by a step-size after every correct answer. This was an effort to avoid an extensive number of trials in which the difference in *f_o_* between the tokens was either too easy or too difficult for participants to discriminate. Following the first incorrect judgment, the procedure changed to a 2-down-1-up TAFC paradigm, in which two correct judgments in a row were required to elicit a reduction in the difference between the base and test token *f_o_* values; a single incorrect judgment still resulted in an increase in the difference between the base and test token *f_o_* values. The run ended after either 10 reversals (i.e., a change in direction of the test token, caused by two correct judgments followed by an incorrect judgment, or an incorrect judgment followed by two correct judgments. See four reversals marked with the red arrows in Figure 1), or when 60 trials had been completed. Using this 2-down-1-up TAFC paradigm allowed for estimation of the participant’s discrimination abilities, located at 70.7% accuracy on the psychometric function [57], hereafter, referred to as the just-noticeable-difference (JND) value. Participants completed either one (CwVN, N = 8; CwTV, N = 19; adults, N = 3) or two (CwVN, N = 8; CwTV, N = 16; adults, N = 17) runs, depending on compliance.

### 2.7. Data Analysis

All statistical analyses were conducted in Minitab [58]. JND values were calculated by averaging the test token *f_o_* values of the last four reversals, reported as the difference in ST of the reversal average referenced to the base token (216.2 Hz) using Equation (1).

(1) $$\mathrm{Semitone}\;\mathrm{conversion}=\frac{12log_{10}\left(\frac{\mathrm{Reversal}\;\mathrm{average}\;(\mathrm{Hz})-216.2\;\mathrm{Hz}}{216.2\;\mathrm{Hz}}\right)}{lo{g_{10}}^{2}}$$

If a participant completed two runs, the JND values from both runs were averaged together. Two estimates of attention were included: The percent of ‘catch’ trials that were judged correctly, and variability of the reversals, defined as the standard deviation of the last four reversals [35,36]. JND values from a given run were not included if the participant answered less than 60% of the catch trials correctly or if they had less than 6 reversals. A total of sixteen participants (12 CwTV, 1 CwVN, and 3 adults) who were included in the analyses had at least one run that was removed due to having less than 60% correct for the catch trials (N = 10), having less than 6 reversals (N = 5), or equipment error during acquisition (N = 1). Of note, an additional ten children that were run on the experimental paradigm were completely excluded from the analysis (and thus, not described in group descriptions, results, or discussion) because they did not have any usable runs. These participants were either younger CwTV children (N = 8, mean 6.5 years, 5.5–8.0 years; two participants had less than sixty percent correct on catch trials, five participants had less than six reversals, and one participant did not understand the training and therefore did not continue to the task), or CwVN (N = 2, mean 7.8 years, 7.1–8.5 years; two participants had less than six reversals; the parent of one of these participants informed the experimenter after the run that the participant had perfect pitch).

#### 2.7.1. Comparison between CwTV and CwVN Groups

To examine hypothesis one, 16 age-matched male CwTV and CwVN were examined for potential differences in pitch discrimination abilities (JND values) and two estimates of attention (the percent of ‘catch’ trials that were judged correctly and the standard deviation of the last four reversals). Three linear regressions were conducted to evaluate whether there were main effects of age or group (CwTV, CwVN), or an interaction of age and group for either pitch discrimination abilities or estimates of attention

#### 2.7.2. Comparison among Younger CwTV, Older CwTV, and Adult Groups

To evaluate hypothesis two, potential group (younger CwTV, older CwTV, adult) differences among pitch discrimination abilities (JND values) and two estimates of attention (the percent of ‘catch’ trials that were judged correctly and the standard deviation of the last four reversals) were evaluated with three non-parametric Kruskal-Wallis tests. Dunn’s multiple comparison test was used to examine significant group differences, with a family alpha level of 0.2 and a Bonferroni individual alpha level of 0.067.

## 3. Results

### 3.1. Comparison between CwTV and CwVN Groups

**Figure 2.**
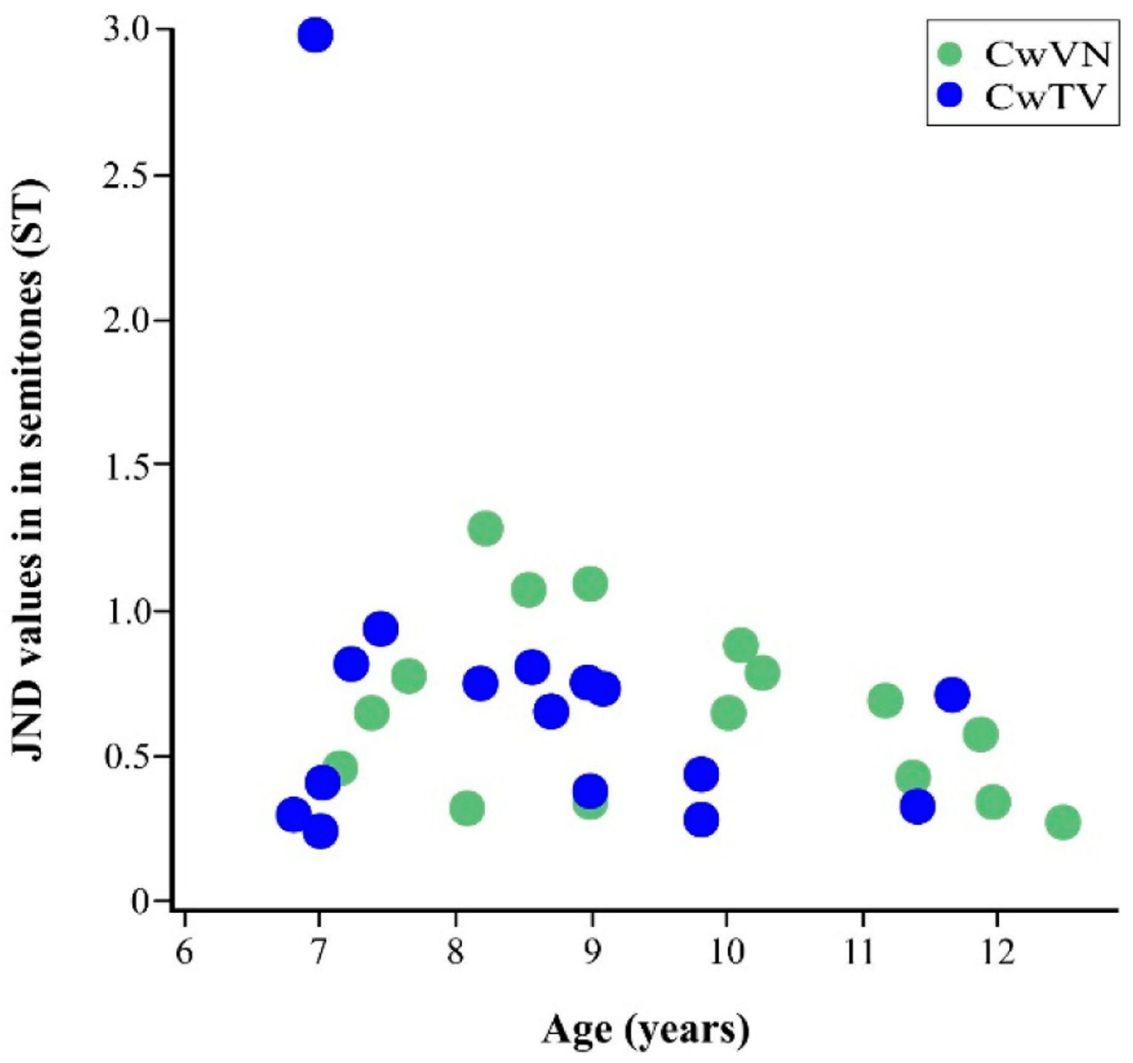
Just-noticeable-difference (JND) values in semitones (ST) for the children with vocal fold nodules (CwVN, green circles) and the age-matched children with typical voices (CwTV, blue circles).

JND values as a function of age and group are displayed in Figure 2. There were no significant main effects of age or group, nor were there any significant interactions of age × group on JND values, the percent of ‘catch’ trials that were judged correctly, or the standard deviation of the last four reversals (all *p* > 0.05). Additionally, the participant with a 2.98 ST JND value (Figure 2) was removed as a potential outlier, and all three regressions were repeated. All factors and interactions continued to be not significant (all *p* > 0.05), thus that subject remained in the dataset.

### 3.2. Comparison among Younger CwTV, Older CwTV, and Adult Groups

**Figure 3.**
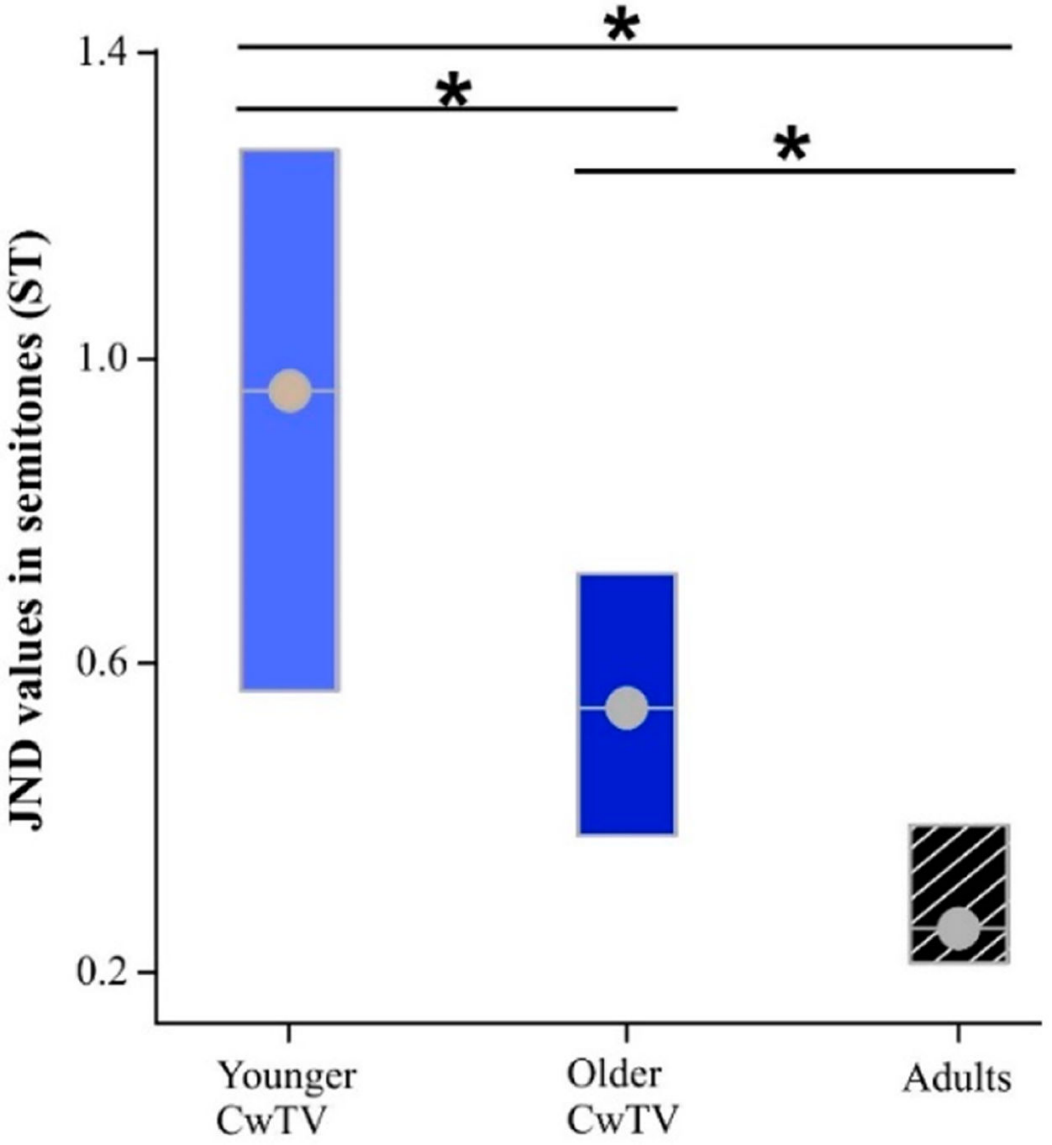
Medians and 95% confidence intervals for the just-noticeable-difference (JND) values in semitones (ST). Markers indicate median JND values; groups that have significantly different medians at *p* < 0.05 are indicated by asterisk. Median confidence interval boxes indicated for the younger children with typical voices (CwTV, light blue), older CwTV (dark blue), and adult (black with white stripes) groups.

Median JND values were significantly different between groups (younger CwTV, older CwTV, and adult, *p* < 0.001). *Post hoc* pairwise comparisons indicated the median JND value for the younger CwTV group (0.96 ST) was significantly larger than both the older CwTV (0 .54 ST) and adult (0.25 ST, both *p* < 0.004) groups (Figure 3). The older CwTV group was also significantly larger than the adult group (*p* = 0.04). Neither estimate of attention (percent coreecf of ‘catch’ trials, standard deviation of last four reversals) was significantly different between groups (*p* > 0.05).

## 4. Discussion

### 4.1. Comparison between CwTV and CwVN Groups

We initially hypothesized that CwVN would have poorer auditory discrimination abilities than CwTV; however, this was not supported by our data. Examination of age-matched CwVN and CwTV indicated that these two groups did not differ based on pitch discrimination abilities. Additionally, estimates of attention were examined in children with and without vocal fold nodules, and there were no significant differences for either estimate of attention between CwTV and CwVN. Thus, although previous work has suggested that CwVN may have attentional differences [31,45,46], these differences appear to manifest at a larger personality-based level that does not translate to the perceptual discrimination of voice. The lack of difference in pitch discrimination abilities is different than what has been noted in adults with dysphonia [33], suggesting potential differences between CwVN and adults with vocal hyperfunction. Heng Tam and colleagues [33] hypothesized that adults with hyperfunction had differences in their discrimination abilities due to an adaptation effect. This suggests that an individual who is consistently listening to their own dysphonic voice may rely less on auditory feedback, as the consistent vocal source they hear is variable. Thereby, this adaptation to a variable mechanism could result in impaired discrimination abilities. The current study hypothesized that CwVN would have poorer discrimination abilities; however, the voices of the CwVN in the current study were not recorded. Therefore, direct relationships cannot be drawn between their dysphonia severity and pitch discrimination abilities. However, it is likely that many CwVN have been dysphonic for less time than an adult with a voice disorder and, thus, may have less of an adaptation effect. Future work examining the relationship between dysphonia severity in CwVN and pitch discrimination abilities is needed to further elucidate this potential connection.

### 4.2. Comparison among Younger CwTV, Older CwTV, and Adult Groups

Pitch discrimination abilities are frequently examined using pure tone stimuli [34-39], with only a few studies examining children’s abilities to discriminate pitch stimuli with complex harmonic structures [40,41]. Results from the current study are consistent with our second hypothesis, as well as previous literature, demonstrating that pitch discrimination abilities improve, and become more adult-like as a function of development [34-39]. Specifically, younger CwTV (under 8 years of age) had significantly higher JND values than both older CwTV and adults, suggesting poorer pitch discrimination abilities in younger CwTV. Additionally, although older CwTV’s pitch discrimination abilities were improved as compared to younger CwTV, they were still significantly poorer than the adult group. Some children from the younger and older CwTV groups were able to achieve adult-like JND values, suggesting JND is not solely related to age. However, at the group level, both younger and older CwTV had immature perceptual abilities, indicating that auditory discrimination of vocal pitch continues to develop in this time-period.

In addition to pitch discrimination abilities, attention was also considered in the current study, as previous researchers have noted that inattention can have meaningful effects on discrimination abilities [35,40]. To address this, the current paradigm was designed to have: (1) A maximum of 60 trials, (2) flexible starting points in order to begin the run closer to an individual’s anticipated discrimination ability, and (3) a 1-down-1-up procedure to assist participants in quickly moving through the initial portion of the paradigm. Additionally, two estimates of attention were examined during the analysis. The first was the percent correct of ‘catch’ trials, wherein the base and test tokens had the same *f_o_*; a lower percent correct value in these trials was considered indicative of decreased attention. The second measure was the standard deviation of the reversals. It has been proposed that when an adaptive stair-step procedure is accurately measuring an individual’s discrimination abilities, there will be lower variability around the JND value; this is contrasted with an individual who is guessing, resulting in higher variability around the JND value (see [35] for additional details). When examining these two estimates of attention in the analysis of JND values, no significant differences between the groups were found for either estimate of attention. Although these findings suggest that inattention was not a significant factor in the increased JND values noted in both younger CwTV and older CwTV, these measures are not a direct measure of attention, but rather estimates tailored to the current study design. Further work would need to examine direct measures of attention, to conclusively determine if different levels of attention impacted the JND values.

### 4.3. Clinical Implications for Voice Therapy in CwVN

Findings from the current study about the pitch discrimination abilities of CwVN as well as CwTV have implications for conducting voice therapy with children. Many classical voice therapy techniques suggest spending extensive time in therapy on attention and awareness (e.g., [32]); however, results from this study and others [1,3] suggest that this may be not a productive use of time. Instead, current models (i.e., [13]) suggest focusing on motor learning principles and ‘direct’ therapy. In a randomized clinical trial that evaluated the ‘direct’ versus ‘indirect’ voice therapy in CwVN, improvements were seen from both therapies [28]. Yet, when evaluating the difference in outcome measures for ‘direct’ as compared to ‘indirect’ therapy across age, larger effect sizes were seen in children over eight years of age. The authors proposed that this difference may be related to the increased maturity and cognitive development in children over the age of eight. Consistent with this timeframe, the current study also showed differences between younger and older children in auditory discrimination abilities. Therefore, if children in the clinical trial [28] over the age of eight had adult-like discrimination abilities, this may have provided them with an increased ability to hear subtle differences in voices. This ability could subsequently allow them to benefit more from direct therapy. As such, when designing voice therapies for children, especially under the age of eight, speech-language pathologists may want to consider the development of auditory discrimination abilities. Future work should continue to examine children with and without vocal fold nodules to understand the development of perceptual, cognitive, and motor abilities in order to apply these directly to the design and implementation of voice therapy tasks.

### 4.4. Limitations and Future Directions

Although this study provided insight into pitch discrimination abilities in children, the small number of total participants means that findings should be interpreted with caution. Furthermore, while the CwTV group did not have vocal complaints, they were not endoscopically screened for the absence of a voice disorder, and some participant’s voices were rated as dysphonic; thus we cannot definitively rule out the presence of a voice disorder. Future work is needed with a larger number of children in the CwVN and CwTV groups to confirm the absence of differences in auditory discrimination abilities. Additionally, hearing screenings were not conducted on the majority of the CwVN group, as well as some participants in the CwTV group. Therefore, we cannot rule out potential auditory threshold differences between the groups in the current study. Although there is some emerging evidence of hearing differences between CwVN and children with voice disorders [59,60], these studies examine a relatively small sample size of children with multiple voice disorders. The hearing differences outlined included both temporary conductive hearing loss and sensory neural hearing loss; therefore, further work in a larger group is needed to elucidate whether CwVN have a higher incidence of hearing difficulties than age-matched peers. Some participants in the current study (in both the CwVN and the CwTV groups) had speech or language deficits. Although including these participants makes this group more representative as a sample of the population, it resulted in less homogenous participant groups. Without directly examining speech, language, and cognition, we cannot say whether these groups were distinct in these areas.

Additionally, participants were recorded in different locations; although all participants were recorded in a quiet area, there may have been an unwanted effect based on participant comfort. Specifically, many CwVN were recorded before a medical examination, potentially increasing their anxiety or distractibility. Future work should control these external and participant factors when examining children with and without vocal fold nodules. Due to the nature of the task, it is possible that younger participants could become fatigued towards the end of a run, which could impact their JND scores. This was unlikely to be an issue given the average run times for both children (5.16 min) and adults (4.17 min); however, future work should evaluate JND values for this groups on a shorter paradigm. Finally, the current work focused on discrimination abilities of vocal pitch, and therefore does not provide information on the perception of other vocal percepts common in CwVN, such as breathiness and strain. Vocal pitch was selected, as it is a salient feature of voice that would not require extensive training to learn, thereby reducing the complexity of the task, and making it appropriate for a range of children. Future work should examine whether discrimination abilities for additional vocal percepts differ between CwVN and CwTV.

## 5. Conclusions

Pitch discrimination abilities were significantly poorer in CwTV younger than eight years of age as compared to older CwTV and adults. Additionally, pitch discrimination abilities continue to be poorer in older children compared to adults. These results suggest that pitch discrimination abilities are still developing, and although the older children were approaching adult-like values, both older and young children continue to have immature perceptual abilities. There were no differences in pitch discrimination abilities between CwTV and CwVN. Additionally, estimates of attention did not differ by age in CwTV, or when comparing CwTV and CwVN groups. Results from this study indicate that CwVN do not have a deficit in auditory discrimination abilities that would preclude them from learning targets during voice therapy. However, the developmental pattern noted for the maturation of pitch discrimination abilities should be considered when designing voice therapy tasks and targets for CwVN.
